# Chemical Compatibility of n-Type Dopants for SWCNT Cathodes in Inverted Perovskite Solar Cells

**DOI:** 10.3390/nano16010064

**Published:** 2026-01-01

**Authors:** Achmad Syarif Hidayat, Naoki Ueoka, Hisayoshi Oshima, Yoshimasa Hijikata, Yutaka Matsuo

**Affiliations:** 1 Department of Chemical Systems Engineering, Graduate School of Engineering, Nagoya University, Nagoya 464-8603, Aichi, Japan; achmad.syarif.hidayat.w3@s.mail.nagoya-u.ac.jp (A.S.H.); ueoka.naoki.z1@f.mail.nagoya-u.ac.jp (N.U.); 2 Institute of Materials Innovation, Institutes of Innovation for Future Society, Nagoya University, Nagoya 464-8601, Aichi, Japan; oshima.hisayoshi.c3@f.mail.nagoya-u.ac.jp; 3 Advanced Research and Innovation Center, Denso Corporation, Kariya 448-8661, Aichi, Japan; yoshimasa.hijikata.j7t@jp.denso.com

**Keywords:** inverted perovskite solar cells, interfacial stability, n-type doping, single-walled carbon nanotubes

## Abstract

The advancement of efficient and stable perovskite solar cells (PSCs) increasingly depends on developing flexible, metal-free electrode architectures. Single-walled carbon nanotubes (SWCNTs) offer chemical robustness, high conductivity, and mechanical flexibility, making them promising candidates to replace brittle metal cathodes. However, pristine SWCNTs are intrinsically p-type, creating energy barriers and recombination losses in inverted (p–i–n) PSCs. Achieving stable n-type doping compatible with both SWCNTs and perovskites is therefore critical. Here, seven representative n-type dopants, small molecules (TBD and TPP), ionic salts (TBAI, TBABr, and B18C6·KCl), and polymers (PEI and PVP) were systematically investigated to elucidate their effects on doping efficiency and interfacial stability. Morphological, structural, and electronic analyses supported by DFT calculations reveal that strong bases and ionic dopants promote perovskite degradation, whereas polymeric and coordination-type dopants preserve crystallinity and surface uniformity. Among them, PEI- and TPP-doped SWCNT electrodes achieved the best device performance, with power conversion efficiencies of 9.6% and 8.1%, respectively, demonstrating efficient electron extraction and interfacial stability. These findings highlight that interfacial chemical compatibility rather than intrinsic donor strength governs the effectiveness of n-type SWCNT doping, providing rational design principles for stable, metal-free perovskite photovoltaics.

## 1. Introduction

The endless pursuit of efficient and stable perovskite solar cells (PSCs) increasingly emphasizes the development of metal-free and flexible electrode architectures [1,2,3,4]. Conventional electrodes, such as indium tin oxide (ITO) and metal, suffer from brittleness and high cost and are prone to ion migration that catalyzes perovskite degradation under operational conditions [5,6,7,8,9,10]. Carbon-based materials, particularly single-walled carbon nanotubes (SWCNTs), have attracted growing attention as sustainable alternatives owing to their chemical stability [11,12,13,14]. Moreover, their solution processability and mechanical robustness make them well suited for advancing printable and flexible perovskite photovoltaics [15]. However, the control of carrier type in SWCNTs is crucial for their integration into diverse optoelectronic and energy-related devices. In their pristine state, SWCNTs generally exhibit p-type semiconducting behaviour when exposed to ambient air, caused by oxygen adsorption inducing hole-like carrier properties [16,17,18,19]. This intrinsic p-type nature has enabled their use in applications such as hole-transporting layers, transparent anodes [18,20,21,22,23,24,25,26,27,28], and p-type field-effect transistors (FET) [29,30,31,32,33,34,35]. However, many emerging carbon-based devices require n-type SWCNTs to enable complementary or ambipolar operation [36,37,38]. Thus, stable and compatible n-type doping has become a key research direction, particularly into PSC devices, as the n-type dopant must be simultaneously compatible with both the SWCNT and the perovskite layers. In inverted (p–i–n) PSC configurations, the requirement for an n-type SWCNT as a cathode becomes even more critical. Pristine p-type SWCNTs create a large energy barrier at the interface, leading to charge accumulation and non-radiative recombination losses. n-type doping is required to reduce the SWCNT work function, enhance electron selectivity, and improve open-circuit voltage and fill factor [39]. While significant progress has been made in the molecular doping of carbon nanomaterials, establishing their chemical compatibility for PSC application remains unexplored.

**Figure 1:**
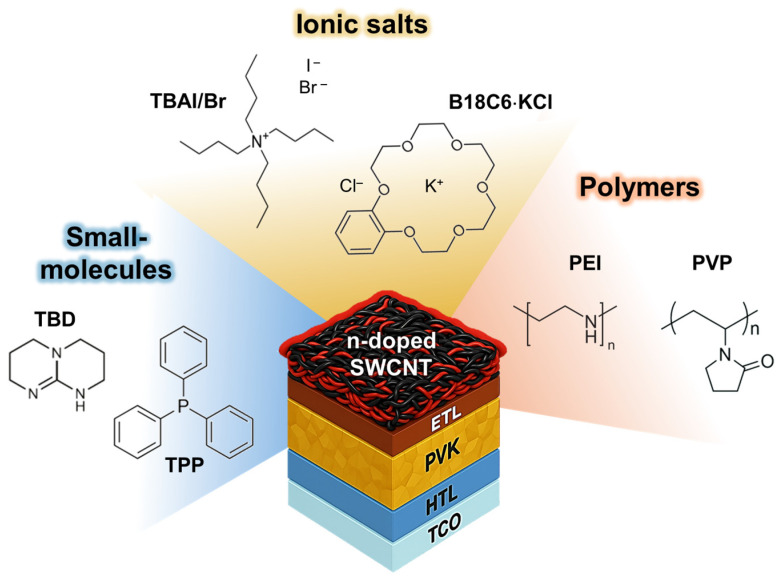
Chemical structures and classification of seven representative n-type dopants for SWCNT cathodes in inverted perovskite solar cells, categorized into three groups: small molecules (blue), ionic salts (yellow), and polymers (orange).

In this work, we systematically investigate the interfacial compatibility and doping behavior of seven representative n-type dopants for SWCNT cathodes in inverted perovskite solar cells (Figure 1). n-type dopants with three different chemical classes, such as small-molecule donors (1,5,7-triazabicyclo [4.4.0]dec-5-ene (TBD), triphenylphosphine (TPP)), ionic salts (tetrabutylammonium iodide (TBAI), tetrabutylammonium bromide (TBABr), and benzo-18-crown 6-ether KCl (B18C6·KCl)), and polymeric dopants (polyethylenimine (PEI) and polyvinylpyrrolidone (PVP)) were employed. These dopants were selected to represent diverse electron-donating mechanisms, Lewis basicity, ionic charge transfer, and coordination-assisted or polymer-mediated doping, allowing systematic evaluation of their structural influence on electronic modulation and perovskite compatibility. To elucidate their effects, we conducted comprehensive morphological (SEM), structural (XRD), and electronic (photoelectron yield spectroscopy, sheet resistance, and Seebeck coefficient) analyses, supported by density functional theory (DFT) calculations of frontier molecular orbitals. The results show that strong bases and ionic dopants (TBD, TBABr, TBAI, and B18C6·KCl) promote perovskite degradation through halide exchange and surface corrosion, as evidenced by the formation of PbI_2_, their hydrated intermediate, and pinholes with roughened film morphology. In contrast, polymeric and coordination-type dopants (PEI, PVP, and TPP) preserve surface uniformity and crystallinity, further enabling moderate Fermi-level tuning and favourable interfacial stability. Devices employing chemically compatible dopants, particularly PEI and TPP, exhibit the highest power conversion efficiencies and interfacial stability, with power conversion efficiencies of 9.6% and 8.1%, respectively. Collectively, these findings demonstrate that interfacial, chemical, and electronic compatibility rather than intrinsic donor strength governs the effectiveness of n-type SWCNT doping, offering rational design guidelines for stable, metal-free perovskite photovoltaics.

## 2. Materials and Methods

### 2.1. Synthesis and Characteristics of SWCNT Films

SWCNT films were synthesized via floating-catalyst chemical vapor deposition (FCCVD) using methane (CH_4_) as the carbon source. Sulfur vapor (promoter) was supplied by flowing N_2_ through a sulfur reservoir maintained at 60 °C, and ferrocene vapor (catalyst precursor) was supplied by flowing N_2_ through a ferrocene reservoir maintained at 30 °C. The carrier gas was N_2_ containing 5% H_2_, with a total flow rate of 1000 sccm applied during both the temperature ramping and growth or collection stages. The reactor temperature was increased to the growth temperature at a ramping rate of 30 °C min^−1^, and SWCNT growth was performed at 950 °C. The SWCNT film collection time was 30–2400 min, adjusted depending on the target collection area and film transparency. The resulting SWCNTs were collected on mixed cellulose ester membranes (RAWP14250, Merck Millipore, Darmstadt, Germany). Film transparency (35–40%) and sheet resistance (35–50 Ω sq^−1^) were adjusted by collection time (Figure S1a). Raman analysis confirmed high-quality SWCNTs with diameters of 1.4–1.7 nm and an *I*_D_/*I*_G_ ratio of 0.007 (Figure S1b).

### 2.2. Perovskite Solar Cell Fabrication

Glass/ITO substrates (15 × 15 mm^2^, 10 Ω/sq; Techno Print Co., Ltd., Fujimino, Japan) were cleaned via UV/O_3_ treatment for 30 min to remove residual organic contaminants. A PEDOT:PSS layer (Clevios P VP, Heraeus Precious Metals GmbH & Co., Hanau, Germany) was prepared by filtering through a 0.2 µm syringe filter (JP020AN, ADVANTEC, Tokyo, Japan), spin-coated at 3000 rpm for 20 s, and annealed at 115 °C for 10 min. MAPbI_3_ perovskite films were fabricated inside a glovebox by dissolving 355 mg PbI_2_ (TCI, Tokyo, Japan) and 122 mg MAI (Sigma Aldrich, St. Louis, MO, USA) in 490 µL DMF (Wako), followed by the addition of 55 µL DMSO (Wako, Osaka, Japan). The precursor solution was spin-coated at 4000 rpm for 20 s, with 150 µL chlorobenzene (Sigma Aldrich, St. Louis, MO, USA) dripped 7 s after spin start. The substrate was then heated at 100 °C for 10 min to form a dark brown MAPbI_3_ layer. 30 mg/mL PCBM (Frontier Carbon Corp., Tokyo, Japan) in chlorobenzene was spin-coated at 4000 rpm for 20 s. SWCNT films were then press-transferred onto the PCBM layer inside the glovebox, followed by spin coating (at 2000 rpm, 30 s) the n-type dopants (TBD 0.5 wt%, TBAI, TBABr 1 wt%, B18C6·KCl 1.5 wt%, PEI, PVP 1 wt%) dissolved in super dehydrated isopropanol (Wako, Osaka, Japan) or (TPP 20 wt%) in deoxidized chlorobenzene (TCI, Tokyo, Japan). According to our previous study, since CB can partially dissolve or redistribute PCBM, an additional PCBM overlayer was applied only for the TPP-doped sample to complete the device structure [39]. For the undoped reference, the solvent alone was applied without dopants. Finally, 100 nm Ag was thermally evaporated only at the device edges to secure electrical contacts for reliable *J*–*V* measurements.

### 2.3. Characterization

*J*–*V* measurements were performed using a software-controlled source meter (Keithley 2400) at a scan rate of 50 mV s^−1^ under simulated AM 1.5 G illumination (100 mW cm^−2^, HAL-320 W, Asahi Spectra, Tokyo, Japan) equipped with a 300 W Xe lamp. The irradiance was calibrated before each measurement using a silicon pyranometer (ML-01, EKO Instruments Co., Ltd., Tokyo, Japan). All measurements were conducted outside the glovebox with a 0.04 cm^2^ mask defining the active area. Surface morphologies were observed by means of scanning electron microscopy (SEM; JSM-7000F, JEOL, Tokyo, Japan). All SEM images were acquired under identical accelerating voltage and detector settings to ensure that the contrast differences originate from the samples rather than imaging conditions. Atomic force microscopy observation was carried out with NX20 AFM (Park Systems, Suwon, Republic of Korea). X-ray photoelectron spectroscopy (XPS) spectra were acquired using ESCALAB250 (Thermo VG Scientific, Grinstead, UK). Baselines were first subtracted using a Shirley background, and peaks were fitted with Gaussian functions in Origin. Raman spectra were taken using a confocal Raman microscope system (inVia reflex, Renishaw, Gloucestershire, UK), using a 532 nm excitation laser and 1800 L/mm grating. Work functions were measured by photoemission yield spectroscopy in air (PYSA; AC-2, Riken Keiki, Tokyo, Japan). Raman spectra were obtained using a confocal Raman microscope (inVia Reflex, Renishaw) with a 532 nm excitation laser and 1800 L/mm grating. X-ray diffraction (XRD; RINT-2500TTR, Rigaku Holdings Corp., Tokyo, Japan) using Cu K_α_ radiation (*λ* = 0.15456 nm) was employed to analyze the crystal structure. Sheet resistance was determined using a four-probe back-contact method. Seebeck coefficients (*S*) were measured using a ZEM-2 system (Advance Riko, Yokohama, Japan) under a helium atmosphere. A temperature gradient (Δ*T* = 10, 15, 20 °C) was applied by heating one side of the substrate to 130 °C, with two K-type thermocouples placed 0.6 cm apart. The thermovoltage (Δ*V* = *V*_cold_ − *V*_hot_) was recorded simultaneously, and *S* = Δ*V*/Δ*T* was calculated after correcting for the contact wire contribution (22.7 µV K^−1^).

### 2.4. Computational Calculation Method

All dopant molecules were optimized using Gaussian 16 with DFT at the B3LYP/6-31G level with frequency analysis. Molecular electrostatic potential (MESP) maps were generated to visualize charge distribution and electron-rich regions. For ionic salts, electron affinity (EA) was calculated from the energy difference between neutral and anionic states. The obtained HOMO levels, EA values, and MESP distributions were used to evaluate the n-type doping potential with SWCNTs. Molecular structures and MESP maps were visualized using GaussView 6.

## 3. Results

### 3.1. Morphological and Structural Analysis

**Figure 2:**
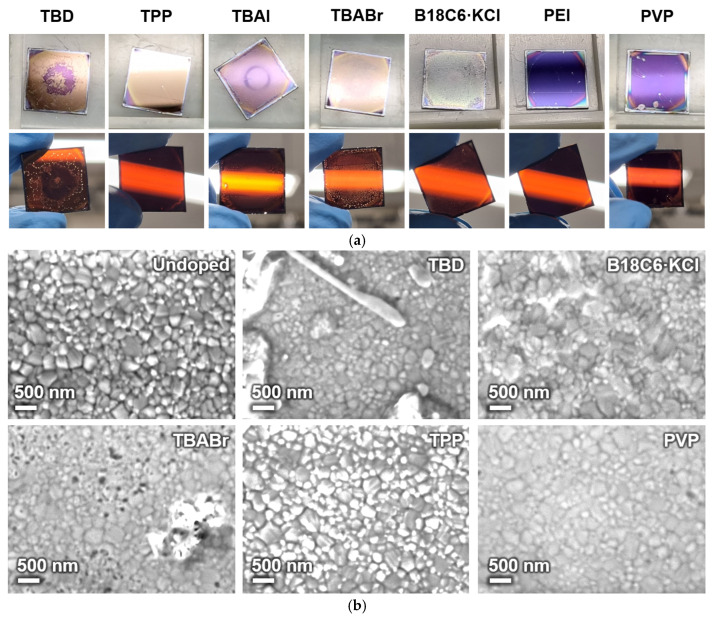
(**a**) Surface and transmission photograph and (**b**) surface SEM images of dopant-treated glass/ITO/PEDOT:PSS/MAPbI_3_/PCBM substrates.

The surface and transmission appearances of dopant-treated glass/ITO/PEDOT:PSS/MAPbI_3_/PCBM substrates are shown in Figure 2a. After dropping the dopant solutions, distinct differences in color and uniformity were observed depending on the dopant type. Samples treated with ionic salts or strongly basic dopants (TBAI, TBABr, B18C6·KCl, and TBD) exhibited irregular surface contrast and visible patchiness with pinholes, suggesting local reactions or surface roughening of the underlying PCBM/perovskite layers. In contrast, samples treated with polymeric (PEI, PVP) or coordination-type (TPP) dopants maintained smooth and uniform surfaces without apparent large-area circular nonuniformity, indicating superior interfacial compatibility.

Surface SEM images of the substrates are shown in Figure 2b. The pristine surface exhibited densely packed perovskite grains with a homogenous layer, indicating uniform film coverage. Upon doping, noticeable morphological changes appeared depending on the dopant species. Samples treated with ionic dopants (e.g., TBABr and B18C6·KCl) displayed irregular granular morphology, pinholes, and partial aggregation, implying interfacial reactions or local dissolution. More severely, the TBD-doped sample exhibited elongated crystallites of 5–10 µm, implying surface re-crystallization or phase segregation (Figure S2). In contrast, polymeric and coordination-type dopants preserved the uniform granular structure with minimal surface damage, indicating more moderate interfacial interaction and better preservation of the interfacial junction. For the TPP-treated sample, the increased bright-contrast regions are attributed to local variations in surface composition and charge dissipation. TPP-rich areas are less conductive, exhibiting enhanced brightness due to localized electron charging during SEM observation.

AFM was further employed to evaluate nanoscale surface uniformity after dopant deposition on the glass/ITO/PEDOT:PSS/MAPbI_3_/PCBM layer (Figure S3). The reference film exhibits a relatively uniform topography with root-mean-square roughness (R_q_) ~6.3 nm. After treatment with polymeric dopants (PEI), similar surface features remain with slightly increased R_q_ to 12.5 nm, indicating that these dopants preserve the underlying structure. In contrast, the TBAI or TBD-treated sample shows increased topographic nonuniformity with significantly increased R_q_ to 45.2–92.0 nm, governing distinct protrusions, most likely salt crystals (90–190 nm height), consistent with the SEM observation.

**Figure 3:**
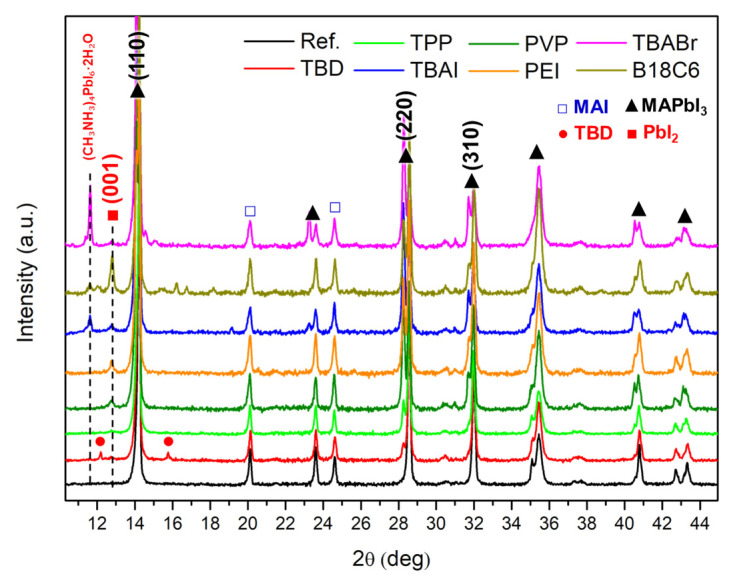
XRD spectra of MAPbI_3_/PCBM layers treated with various dopants.

Figure 3 shows X-ray diffraction (XRD) patterns of doped MAPbI_3_/PCBM substrates. The undoped substrate exhibited prominent reflections at 14.1°, 28.7°, and 31.8°, corresponding to the (110), (220), and (310) planes of the tetragonal MAPbI_3_ phase. Weak peaks at 20.1° and 24.5° were also detected, likely originating from trace residual MAI, suggesting near-complete but not full conversion of precursors [40]. Upon exposure to various dopant solutions, noticeable variations in peak intensity and the emergence of additional reflections were observed, suggesting structural modifications. In samples treated with ionic dopants such as TBAI, TBABr, and B18C6·KCl, new diffraction features appeared around 11.6° and 12.6°, which can be assigned to hydrated intermediate phases such as (CH_3_NH_3_)_4_PbI_6_·2H_2_O or CH_3_NH_3_PbI_3_·H_2_O and the (001) plane of PbI_2_, respectively, indicating simultaneous hydration and decomposition via ion exchange in the lattice (1) [41]. Moreover, the observed splitting and shift of the tetragonal (200) peak toward higher angles are indicative of the formation of mixed-halide perovskites (Figure S4) [42].

CH_3_NH_3_PbI_3_ + H_2_O + X^−^ → (CH_3_NH_3_)_4_PbI_6_·2H_2_O + PbI_2_ + CH_3_NH_3_X (X^−^ = Cl^−^, Br^−^, or I^−^) (1)

A distinct behaviour was observed for the TBD-treated sample, where new peaks appeared around 12.1° and 15.7°, attributed to secondary crystallization induced by the strong basicity of TBD. This base promotes CH_3_NH_3_^+^ deprotonation and the formation of TBDH^+^I^−^ complexes (2), leading to localized recrystallization, consistent with the large surface crystallites observed in SEM. Other strong bases and amine-type additives have been shown to chemically interact with cations in halide perovskites [43,44,45].

CH_3_NH_3_^+^I^−^ + TBD → CH_3_NH_2_ + TBDH^+^I^−^ (2)

In contrast, polymeric dopants (PEI and PVP) caused only weak PbI_2_-related peaks with no hydrated intermediate phases, while the main MAPbI_3_ reflections remained largely intact. The coordination-type dopant (TPP) fully preserved the characteristic perovskite diffraction pattern without introducing new peaks, confirming its excellent interfacial compatibility and chemical inertness. These findings highlight that dopant chemistry critically determines structural stability, where soft, electronically compatible dopants (TPP, PEI, and PVP) maintain crystallinity, whereas reactive ionic (TBA, TBAI, and B18C6·KCl) or strongly basic (TBD) species promote phase decomposition and interfacial degradation.

**Figure 4:**
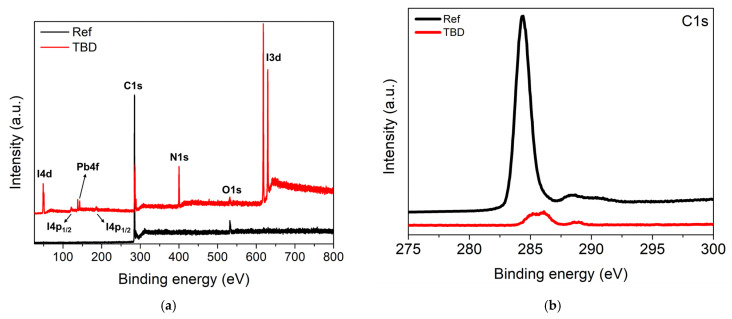

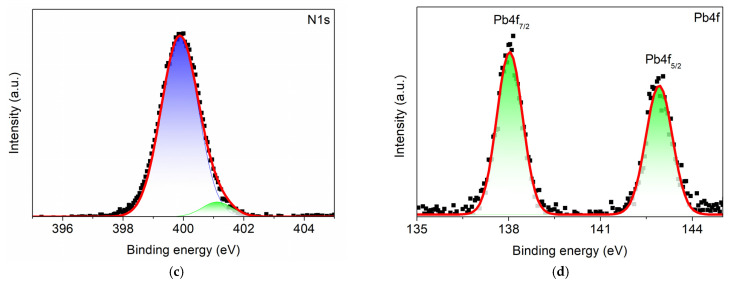
(**a**) Surface XPS survey spectra of ITO/PEDOT:PSS/MAPbI_3_/PCBM before (Ref) and after (TBD) treatment and their corresponding (**b**) C1s, (**c**) N1s, and (**d**) Pb4f spectra. Square markers indicate the raw data, and the red line represents the fitted spectra. In the N1s spectrum, two deconvoluted components are shown, representing neutral (blue) and cationic or protonated (green) nitrogen species. In the Pb4f spectrum, the Pb4f_7/2_ and Pb4f_5/2_ components are highlighted in green.

To further probe the chemical nature of the interface modification induced by TBD, XPS was performed on the ITO/PEDOT:PSS/MAPbI_3_/PCBM surface before (Ref) and after (TBD) coating (Figure 4a). In the reference sample, Pb4f and I3d were below detection, indicating that the measured surface is dominated by the PCBM overlayer. After TBD coating, the C1s peak significantly reduced while Pb4f and I3d became pronounced, together with the appearance of an N1s signal, confirming dopant adsorption and a modified surface (Figure 4b–d). The N1s spectrum can be fitted with two components, a lower-binding-energy peak at ~399 eV attributed to neutral TBD and a higher-binding-energy component at ~401 eV assigned to cationic or protonated TBD (TBDH^+^) (Figure 4c). The emergence of the TBDH^+^ component is consistent with acid–base interaction in the perovskite-related interface, supporting the formation of an iodide-associated salt species, as suggested by Equation (2) [46,47,48]. In parallel, the Pb4f region (~138 and ~142 eV) is dominated by Pb^2+^-type species (Figure 4d) [49]. The combined Pb4f, I3d, and N1s results support TBD-induced interfacial modification consistent with the additional diffraction features observed in the XRD spectra of the TBD-treated sample.

### 3.2. Electrical and Optical Properties

**Figure 5:**
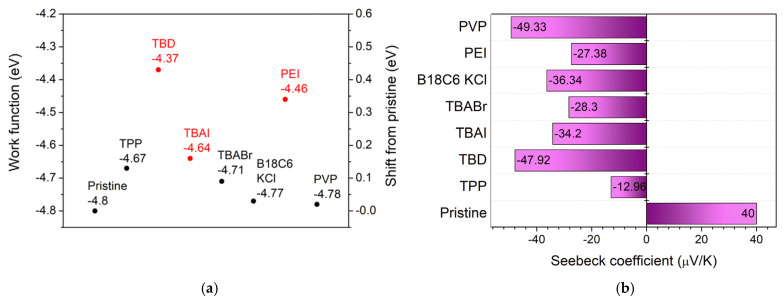
(**a**) Work function and (**b**) Seebeck coefficient of pristine and doped SWCNT films.

The electrical transport behavior of the SWCNT films was evaluated through sheet resistance (*R*_sheet_) (Figure S5), work function (*Φ*) (Figure 5a and Figure S6), and Seebeck coefficient (*S*) (Figure 5b and Figure S7) measurements. The pristine SWCNT film exhibited p-type characteristics with *S* = 40 µV K^−1^, *Φ* = –4.8 eV, and *R*_sheet_ = 35–50 Ω sq^−1^. Upon introducing n-type dopants, a clear reversal in *S* polarity and systematic modulation of *R*_sheet_ were observed, confirming successful electron doping [39]. Among the ionic dopants, TBAI showed the most pronounced conductivity, where at moderate concentrations (10–20 mM or 1–1.5 wt%), *R*_sheet_ was reduced by approximately 30–40% (21.7 Ω sq^−1^). The Seebeck coefficients shifted from positive to negative values (–34 µV K^−1^), followed by *Φ* shift to –4.64 eV. For small-molecule dopants, TBD, an organic superbase, induced the strongest n-type conversion, where at lower concentration (0.5 wt%) *R*_sheet_ was reduced to 41.7 Ω sq^−1^, achieving *S* = –48 µV K^−1^ followed by the largest *Φ* shift to –4.37 eV. Among polymeric dopants, PEI exhibited moderate n-type doping effects, where the Seebeck coefficients reached –27 µV K^−1^ followed by a significant *Φ* shift to –4.46 eV. However, the *R*_sheet_ remained higher than the pristine value (98 Ω sq^−1^). These findings underscore the critical role of dopant strength, electronic softness, and molecular design in controlling SWCNT electronic properties. While strong bases and ionic dopants enable pronounced n-type conversion, they risk morphological instability. Polymer-based dopants offer good interfacial stability and controllable work function tuning but still face limitations in electrical conductivity due to their insulating backbones. The development of electrically conductive or conjugated polymeric dopants with strong electronic coupling to SWCNT will considerably decrease sheet resistance while maintaining environmental and morphological stability. The experimental trends in work function, Seebeck coefficient, and sheet resistance are consistent with the DFT-calculated and literature-reported electronic parameters of the dopants. Optimized geometries and frontier orbital energies, together with reported electron affinities (EAs), were considered to evaluate their donor–acceptor tendencies (Table S1 and Figure S8). Dopants with higher HOMO levels and lower electron affinities in their chemical class exhibit stronger electron-donating ability, leading to larger upward shifts in *Φ* and smaller-magnitude (milder) negative *S* values.

Raman spectroscopy was used to evaluate SWCNT and doping-induced electronic modulation after dopant treatment (Figure S9). Compared with pristine SWCNTs, all dopant-treated samples showed a downshift of the G band, indicating effective n-type molecular doping. To compare the degree of structural perturbation, the D-band to G-band intensity ratio (*I*_D_/*I*_G_) was quantified (Table S3). The pristine SWCNT film exhibited a low *I*_D_/*I*_G_, indicating high structural quality. TPP showed slightly increased *I*_D_/*I*_G_ (0.0113) while PEI (0.0224) showed a modest increase. In contrast, TBABr (0.0367), TBAI (0.0419), B18C6 (0.0424), TBD (0.0479), and PVP (0.0541) exhibited more pronounced increases, implying stronger perturbation at the SWCNT surface or junctions. These results suggested that the presence of TPP and PEI relatively maintains the structural quality of SWCNT.

Atomic force microscopy (AFM) was performed to examine the nanoscale surface topography of SWCNT films after representative dopant treatments (Figure S10). A 5 × 5 µm^2^ height map was acquired for each sample, and the RMS roughness (Rq) was extracted from the scanned region. The reference SWCNT film showed a Rq of 13.2 nm, while PEI-, TBD-, and TBAI-treated films exhibited Rq values of 13.6 nm, 10.4 nm, and 12.0 nm, respectively. The results suggested dopant-dependent differences in surface coverage within the porous SWCNT network. Slightly higher roughness for PEI suggested that the formation of a polymeric coating at SWCNT bundles and junctions could introduce modest nanoscale height variation. In contrast, the reduced roughness observed for TBD and TBAI suggested partial void filling and infiltration into the SWCNT network followed by bundle compaction during drying, resulting in a smoother apparent surface. None of the dopant treatments caused severe nanoscale roughening compared with the reference, supporting that the SWCNT cathode morphology is largely preserved after doping.

**Figure 6:**
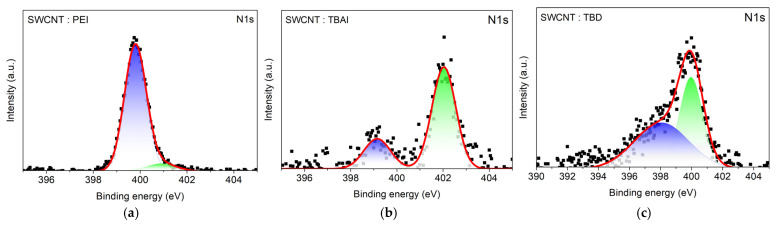
N1s XPS spectra of (**a**) PEI-, (**b**) TBAI-, and (**c**) TBD-doped SWCNT films. Square markers indicate the raw data, and the red line represents the fitted spectra. In the N1s spectra, two deconvoluted components are shown, representing neutral (blue) and cationic or protonated (green) nitrogen species.

XPS survey spectra were collected to confirm dopant incorporation on SWCNT films and to compare dopant-dependent surface composition (Figure S11). After dopant treatment, clear dopant-element signatures appeared, where PEI- and TBD-doped SWCNTs exhibit an N1s signal, confirming adsorption of nitrogen-containing dopants on the SWCNT network, while TBAI-treated SWCNTs show both N1s and I3d peaks, consistent with the presence of tetrabutylammonium iodide at the surface. High-resolution N1s XPS further reveals dopant-dependent nitrogen chemical states on SWCNT films (Figure 6). For PEI-treated SWCNTs, the spectrum can be fitted with two components, a dominant peak at 399.79 eV (92%) assigned to neutral amine-type nitrogen and a minor higher-binding-energy component at 400.9 eV (8%), indicating a small fraction of nitrogen in a more positively polarized or partially ionized environment. In contrast, the TBAI-treated SWCNT film is dominated by a component at 402.03 eV (75%), characteristic of cationic or quaternary ammonium (N^+^), consistent with the ionic nature of tetrabutylammonium at the SWCNT surface. A smaller component at 399.14 eV (25%) suggests an additional nitrogen environment, which may arise from minor neutral species or heterogeneous surface charging upon adsorption. Notably, TBD-treated SWCNTs show two components at 400.0 eV (45%) and 398.1 eV (55%). Almost equal binding-energy contribution indicates a more balanced presence of neutral and cationic ammonium on the SWCNT network. Overall, these distinct N1s chemical-state distributions support that PEI predominantly formed a neutral polymeric modifier on SWCNTs, TBAI introduced ionic N^+^ species at the surface, and TBD exhibited a more balanced mixture of neutral and ionic species.

To further understand the origin of electron transfer and doping behavior, molecular electrostatic potential (MESP) maps were computed for all dopants (Figure S12). The color distribution in the MESP provides insight into charge localization and the ability of each molecule to donate or accept electrons during interaction with SWCNTs. Strongly negative potential regions (red) indicate high electron density, often associated with heteroatoms such as nitrogen, phosphorus, or halides, while positive regions (blue) correspond to electropositive hydrogen or alkyl sites. Ionic dopants show highly localized negative potential around halide anions (−0.186 to −0.227 a.u.), consistent with their large electron affinities and strong driving force for electron transfer to the SWCNT surface. However, their strong ionic character also promotes unwanted interfacial reactions with perovskite layers, as evidenced by XRD, XPS, AFM, and SEM analyses.

In contrast, coordination-type (TPP) and polymeric dopants (PEI and PVP) exhibit more delocalized and small negative potentials across the molecule (−3.2 × 10^−2^ to −7.3 × 10^−2^ a.u.), reflecting their “soft” electronic character and controlled charge donation. This leads to gentle Fermi-level modulation without structural degradation. TBD, possessing an intense localized negative potential on its amidine site, represents an extreme case of strong basicity, favouring rapid electron transfer and MA^+^ deprotonation in perovskites, consistent with its observed interfacial crystallization.

An optimal dopant exhibits a balanced combination of moderate Seebeck coefficient (*S*), low sheet resistance (*R*_sheet_), and an upward work function (*Φ*) shift, signifying efficient and stable n-type conversion of SWCNTs [50]. MESP analysis reveals that dopants with spatially uniform and moderately negative potentials enable gentle and stable electron transfer, preserving interfacial integrity. In contrast, dopants with highly localized negative regions drive stronger but less controlled interactions, often leading to over-doping or instability. These results highlight that balanced electron distribution and chemical softness, rather than strong basicity, dictate effective and durable n-type doping for the SWCNT cathode in inverted PSCs.

### 3.3. Device Performance

**Figure 7:**
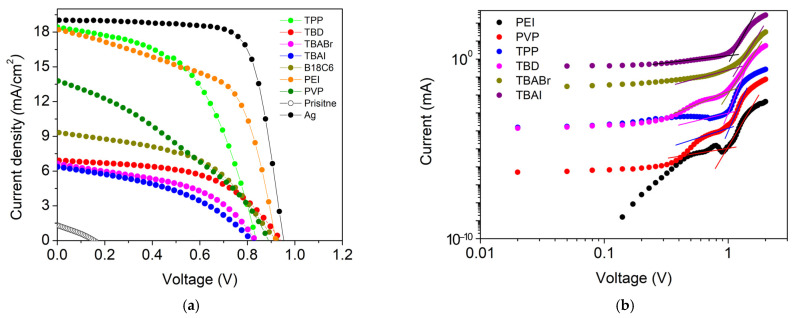
(**a**) *J*–*V* characteristics comparison of inverted perovskite solar cells incorporating different SWCNT cathodes compared with silver (Ag), and (**b**) dark *I*–*V* characteristics (log–log) of electron-only devices (ITO/SnO_2_/MAPbI_3_/PCBM/SWCNT) with different dopant-treated SWCNT cathodes. The trap-filled limit voltage (*V*_TFL_) was extracted from the kink point.

The photovoltaic parameters, *J*–*V* characteristics, and energy level diagram of the inverted perovskite solar cells (ITO/PEDOT:PSS/MAPbI_3_/PCBM/SWCNT:n-dopant) are summarized in Table 1, Figure 7a, and Figure S13, respectively. The pristine SWCNT-based device exhibited very poor performance (PCE = 0.06%) with low *V*_OC_ (0.155 V) and *J*_SC_ (1.33 mA cm^−2^). This confirms that electron-selective transport is severely limited without n-type doping. Upon introducing different dopants, significant variations in photovoltaic performance were observed depending on the dopant class. Devices incorporating ionic dopants (TBAI, TBABr, and B18C6·KCl) showed moderate improvement in *V*_OC_ values (~0.82–0.93 V) but limited *J*_SC_ (6–9 mA cm^−2^) and low fill factors (0.41–0.48), yielding PCEs below 4%. Similarly, the strong base TBD achieved a higher *V*_OC_ (0.94 V) but exhibited low *J*_SC_ (6.92 mA cm^−2^) and a PCE of only 3.46%. Their relatively high *R*_s_ and low *R*_sh_ indicate interfacial recombination and perovskite degradation that causes poor charge extraction.

In contrast, polymeric (PEI and PVP) and coordination-type dopants (TPP) produced markedly better device performance. The PEI-doped SWCNT cathode achieved a PCE of 9.56% (*V*_OC_ = 0.92 V, *J*_SC_ = 18.23 mA cm^−2^, FF = 0.57), representing over a 150-fold improvement compared to pristine SWCNT. The high photocurrent and moderate FF suggest efficient electron extraction and reduced interfacial losses. While maintaining interfacial compatibility, PEI shifts the SWCNT work function to better align with the PCBM LUMO/perovskite, thereby reducing the electron-extraction barrier and supporting efficient charge collection [51]. Similarly, TPP-doped devices exhibited PCE = 8.09%, confirming effective electron extraction while maintaining interfacial stability. PVP yielded moderate performance (PCE = 4.18%), with lower FF attributed to limited conductivity of the polymeric network (Figure S5).

To rationalize the device performance trends, we conducted SCLC measurements using electron-only devices (ITO/SnO_2_/MAPbI_3_/PCBM/SWCNT) with different dopant-treated SWCNT cathodes (Figure 7b). The trap-filled limited voltage (*V*_TFL_) decreases from 1.22 V (TBABr) to 1.09 V (TBAI), 1.03 V (PVP and TBD), 0.99 V (TPP), and 0.96 V (PEI). Because the extracted trap density is related to *V*_TFL_ for identical device thickness, the lower *V*_TFL_ values indicate reduced electron-trap density and improved vertical charge transport or extraction when polymeric (PEI and PVP) and coordination-type (TPP) dopants are used. This provides a consistent explanation for the higher photocurrent observed in devices with polymeric dopants. Although PEI can increase the in-plane sheet resistance of the standalone SWCNT film, it simultaneously lowers trap-assisted losses in the electron-extraction pathway, enabling higher extracted current under operation.

To obtain initial insight into stability, we conducted a shelf-stability test under controlled storage conditions (25 °C, 25% RH, ~300 lux) for devices employing a PEI-doped SWCNT cathode and compared them with Ag cathode devices (Table S2, Figure S14). After 30 days, the PEI-doped SWCNT device retained 7.95% PCE from an initial 9.56% (≈83% retention), with only a small change in *J*_SC_ (18.23 to 17.99 mA cm^−2^) and *V*_OC_ (~0.92 V). The performance loss mainly originates from a reduced FF (0.570 to 0.477) accompanied by decreased *R*_Sh_ (204 to 166 Ω·cm^2^), suggesting increased leakage or recombination. In contrast, the Ag device exhibited a much larger degradation, decreasing from 13.41% to 5.33% (≈40% retention), driven by pronounced losses in *J*_SC_ (19.03 to 13.41 mA cm^−2^), *V*_OC_ (0.954 to 0.864 V), and FF (0.739 to 0.459), together with a substantial increase in *R*_S_ (7.24 to 19.61 Ω·cm^2^) and decrease in *R*_Sh_ (7170 to 1383 Ω·cm^2^). The comparison suggests that the PEI-doped SWCNT cathode can provide improved retention under mild ambient storage relative to Ag.

Overall, these results correlate well with the electronic and morphological analyses. Dopants that balance chemical compatibility and moderate doping strength (TPP, PEI, and PVP) maintain perovskite integrity, whereas ionic and strongly basic dopants induce chemical instability that hinders charge collection. Although the Ag electrode still gives the highest PCE (13.41%), the performance gap has been significantly narrowed, demonstrating that chemically compatible n-type SWCNT cathodes can serve as more stable, metal-free alternatives for inverted perovskite solar cells.

## 4. Conclusions

In this study, we systematically investigated the interfacial compatibility and electronic effects of seven representative n-type dopants, specifically TBD, TPP, TBAI, TBABr, B18C6·KCl, PEI, and PVP, on SWCNT cathodes in inverted perovskite solar cells. The results reveal that the balance between doping strength and chemical compatibility is crucial for achieving stable n-type SWCNTs. Ionic and strongly basic dopants such as TBAI, TBABr, B18C6·KCl, and TBD effectively shift the Fermi level but simultaneously induce perovskite degradation through halide exchange, surface roughening, and MA^+^ deprotonation. In contrast, polymeric (PEI and PVP) and coordination-type (TPP) dopants maintain perovskite crystallinity and interface stability while enabling moderate n-type doping. Electrical characterization and DFT-supported analysis confirm that dopants with spatially distributed electron density and moderate electron-donating strength, demonstrated by PEI and TPP, achieve optimal electronic modulation and minimal interfacial disruption. Devices employing PEI- and TPP-doped SWCNT cathodes reached power conversion efficiencies of 9.6% and 8.1%, respectively, far exceeding the undoped device (0.06%) and approaching metal-electrode performance. Overall, this work establishes a molecular-level framework for designing chemically compatible n-type dopants that balance conductivity, stability, and interfacial integrity, advancing the development of stable, metal-free perovskite photovoltaics employing carbon-based electrodes.

## Figures and Tables

**Table 1:** Electrical properties of inverted PSCs utilizing pristine and doped SWCNT films as a cathode.

Dopants Classification	Dopants	*J*_SC_ (mA/cm^2^)	*V*_OC_ (V)	FF	*R*_S_ (Ω cm^2^)	*R*_Sh_ (Ω cm^2^)	*η* (%)
Small molecules	TPP	18.44	0.838	0.523	12.08	283.84	8.09
	TBD	6.92	0.940	0.531	33.51	875.47	3.46
Ionic salts	TBABr	6.65	0.834	0.464	30.04	326.83	2.58
	TBAI	6.37	0.819	0.412	42.60	310.85	2.15
	B18C6·KCl	9.34	0.927	0.481	35.75	349.19	4.16
Polymer	PEI	18.23	0.920	0.570	7.86	204.31	9.56
	PVP	13.81	0.889	0.340	24.70	144.75	4.18
Reference	SWCNT pristine	1.33	0.155	0.293	58.25	143.09	0.06
	Ag	19.03	0.954	0.739	7.24	7170.3	13.41

## Data Availability

All essential results and data are provided within the article and/or its ESI. Requests for additional information should be addressed to the corresponding authors.

## Supplementary Materials

The following supporting information can be downloaded at https://www.mdpi.com/article/10.3390/nano16010064/s1. Figure S1: (a) Patterned SWCNT films (3 × 8 mm^2^) and (b) their Raman spectrum; RBM modes (100–200 cm^−1^) are shown in the inset; Figure S2: Surface SEM image of TBD-doped glass/ITO/PEDOT:PSS/MAPbI_3_/PCBM substrate; Figure S3: AFM observation of dopant-treated glass/ITO/PEDOT:PSS/MAPbI_3_/PCBM substrates; Figure S4: XRD patterns in the region of the tetragonal (220) peaks (2θ = 28.7°) for the prepared samples; Figure S5: Sheet resistance of SWCNT films treated with various dopants at different doping concentrations; Figure S6: Photoemission yield spectra of pristine and doped SWCNT films; the work function values were determined from the threshold onset of photoemission; Figure S7: Thermoelectric voltage (Δ*V*) versus applied temperature difference (*ΔT*) of pristine and doped SWCNT films; Seebeck coefficients (S) were obtained from the linear slope of the Δ*V*/Δ*T* plots and corrected for contact wire contributions from the instrument (22.7 µV K^−1^); Figure S8: DFT-calculated electronic parameters of the investigated dopants, including HOMO energy levels of molecular dopants and electron affinity from ionic dopants [52]; the PEI and PVP models were represented by diethylenetriamine (DETA) and N-methyl-2-pyrrolidone (NMP) molecules, respectively, to reflect their dominant functional units and electronic characteristics; Figure S9: Raman spectra of pristine and doped SWCNT films; D- and G-band regions are shown for comparison; Figure S10: AFM images of pristine and doped SWCNT films (PEI, TBD, and TBAI) and their corresponding root-mean-squared roughness (RMS, R_q_); Figure S11: Survey XPS spectra of doped SWCNT films (PEI, TBD, and TBAI) labelled against their corresponding elemental compositions; Figure S12: Molecular electrostatic potential (MESP) maps of the investigated dopants; the red and blue regions represent electron-rich and electron-deficient sites, respectively, indicating potential locations for charge transfer interactions with SWCNTs; Figure S13: Energy level diagram of complete device stack incorporating pristine and doped SWCNT films; Figure S14: *J*–*V* characteristics comparison of inverted perovskite solar cells incorporating PEI-doped SWCNT cathodes compared with silver (Ag); Table S1: XYZ Cartesian coordinate from optimized structure of the n-type dopants; Table S2: Electrical properties of inverted PSCs utilizing PEI-doped SWCNT film compared with Ag as cathode; Table S3: D-to-G band intensity ratio (*I*_D_/*I*_G_) of pristine and dopant-treated SWCNT films extracted from Raman spectra.

## Abbreviations

The following abbreviations are used in this manuscript:

SWCNT	Single-Walled Carbon Nanotube
MAPbI_3_	Methylammonium Lead Iodide
CH_3_NH_3_I/MAI	Methylammonium Iodide
PCBM	[6,6]-Phenyl-C_61_-butyric acid methyl ester
PEDOT:PSS	Poly(3,4-ethylenedioxythiophene):Poly(styrenesulfonate)
PEI	Polyethylenimine
PVP	Polyvinylpyrrolidone
TPP	Triphenylphosphine
TBD	1,5,7-Triazabicyclo [4.4.0]dec-5-ene
TBAI	Tetrabutylammonium Iodide
TBABr	Tetrabutylammonium Bromide
B18C6·KCl	Benzo-18-crown-6 potassium chloride complex
PbI_2_	Lead(II) Iodide
DMF	N,N-Dimethylformamide
DMSO	Dimethyl Sulfoxide
CB	Chlorobenzene
ITO	Indium Tin Oxide
Ag	Silver
DETA	Diethylenetriamine
NMP	N-Methyl-2-pyrrolidone
SEM	Scanning Electron Microscopy
XRD	X-ray Diffraction
XPS	X-ray Photoelectron Spectroscopy
AFM	Atomic Force Microscopy
DFT	Density Functional Theory
MESP	Molecular Electrostatic Potential
PYSA	Photoemission Yield Spectroscopy in Air
J–V	Current Density–Voltage
EA	Electron Affinity
HOMO	Highest Occupied Molecular Orbital
LUMO	Lowest Unoccupied Molecular Orbital
PSC	Perovskite Solar Cell
PCE	Power Conversion Efficiency
*V*_oc_	Open-Circuit Voltage
*J*_sc_	Short-Circuit Current Density
FF	Fill Factor
*R*_sheet_	Sheet Resistance
*R*_sh_	Shunt Resistance
*R*_q_	Root-mean-squared Roughness
*V*_TFL_	Trap Filled Limited Voltage
*H*	Efficiency
*S*	Seebeck Coefficient
*Φ*	Work Function
Δ*V*	Thermoelectric Voltage Difference
Δ*T*	Temperature Difference
