# Supplementary material for: Chemical Compatibility of n-Type Dopants for SWCNT Cathodes in Inverted Perovskite Solar Cells
Source: Nanomaterials (Basel). 2026 Jan 1;16(1):64. doi: 10.3390/nano16010064 (PMC12787565; doi:10.3390/nano16010064)
Supplement: Supplementary file 1 [file nanomaterials-16-00064-s001.zip › nanomaterials-4038572-supplementary.pdf]

Chemical Compatibility of n-Type Dopants for SWCNT Cathodes in Inverted Perovskite Solar Cells

Achmad Syarif Hidayat [1], Naoki Ueoka [1], Hisayoshi Oshima [2], Yoshimasa Hijikata [3], and Yutaka Matsuo [1,2,*]

[1] Department of Chemical Systems Engineering, Graduate School of Engineering, Nagoya University, Nagoya, Aichi, Japan; achmad.syarif.hidayat.w3@s.mail.nagoya-u.ac.jp (A.S.H); ueoka.naoki.z1@f.mail.nagoya-u.ac.jp (N.U.)

[2] Institute of Materials Innovation, Institutes of Innovation for Future Society, Nagoya University, Nagoya, Aichi, Japan; oshima.hisayoshi.c3@f.mail.nagoya-u.ac.jp (H.O.)

[3] Advanced Research and Innovation Center, DENSO CORPORATION, Kariya, Aichi, Japan; yoshimasa.hijikata.j7t@jp.denso.com (Y.H.)

* Correspondence: matsuo.yutaka.h7@f.mail.nagoya-u.ac.jp

Supporting Information

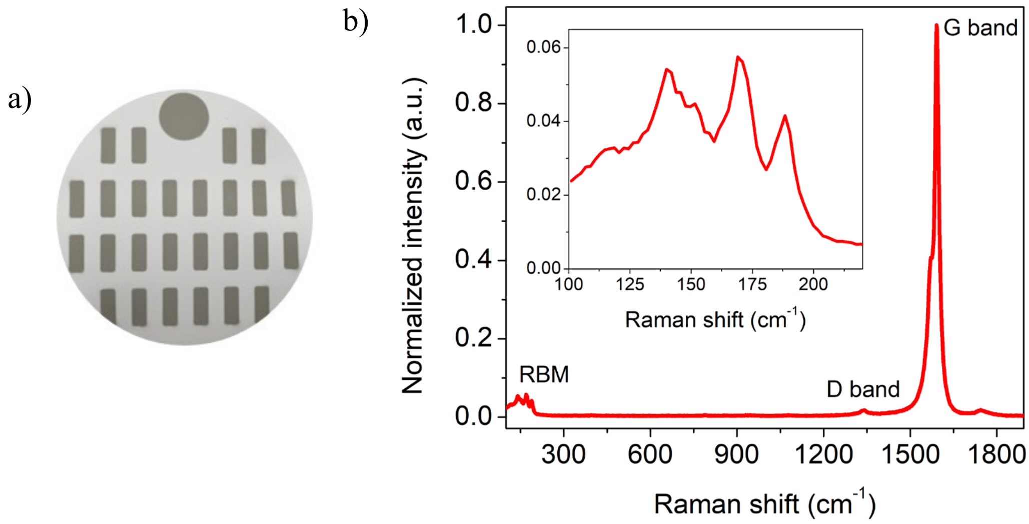

Figure S1 (a) Patterned SWCNT films (3 × 8 mm^2) and (b) their Raman spectrum. RBM modes (100 – 200 cm^{-1}) are shown in the inset.

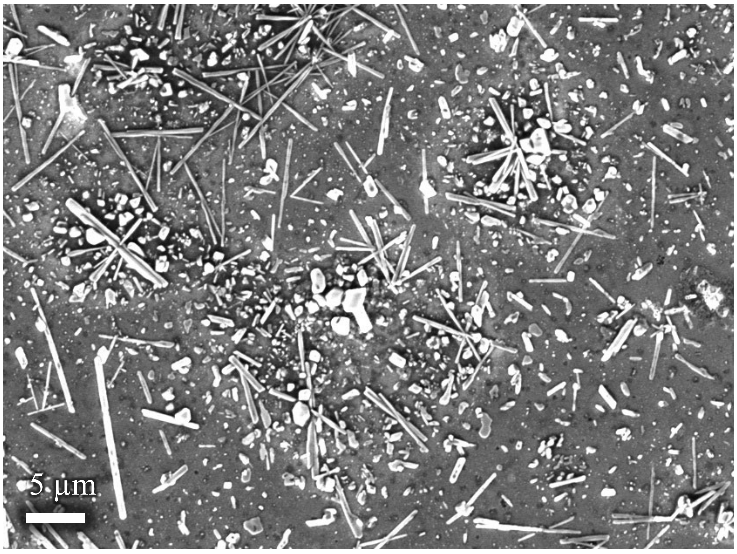

Figure S2 Surface SEM image of TBD doped glass/ITO/PEDOT:PSS/$MAPbI_3$/PCBM substrate.

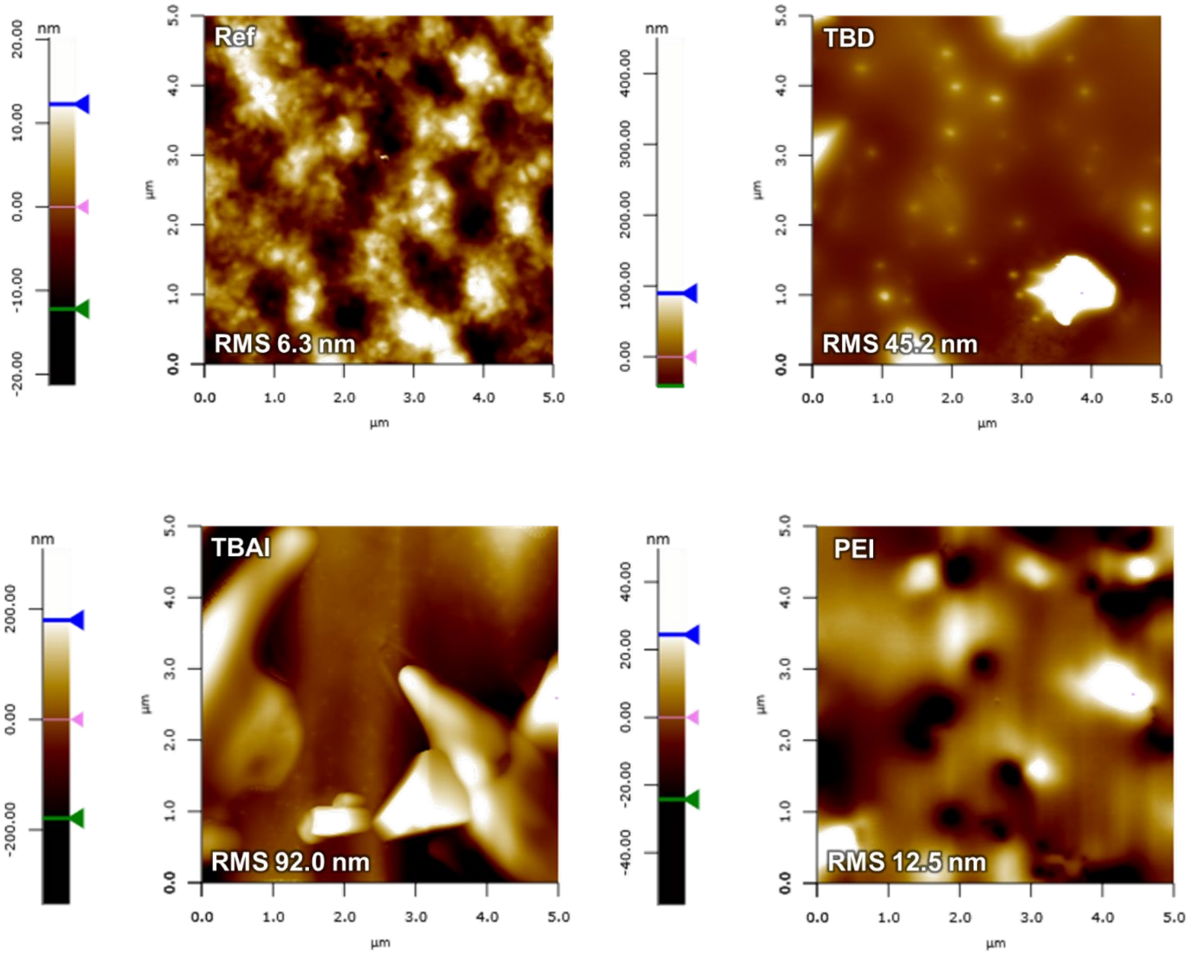

Figure S3 AFM observation of dopant-treated glass/ITO/PEDOT:PSS/$MAPbI_3$/PCBM substrates.

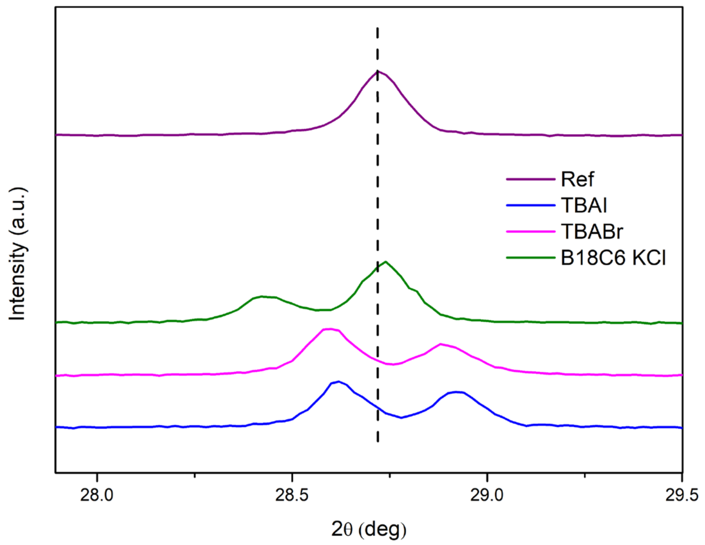

Figure S4 XRD patterns in the region of the tetragonal (220) peaks (2θ=28.7°) for the prepared samples.

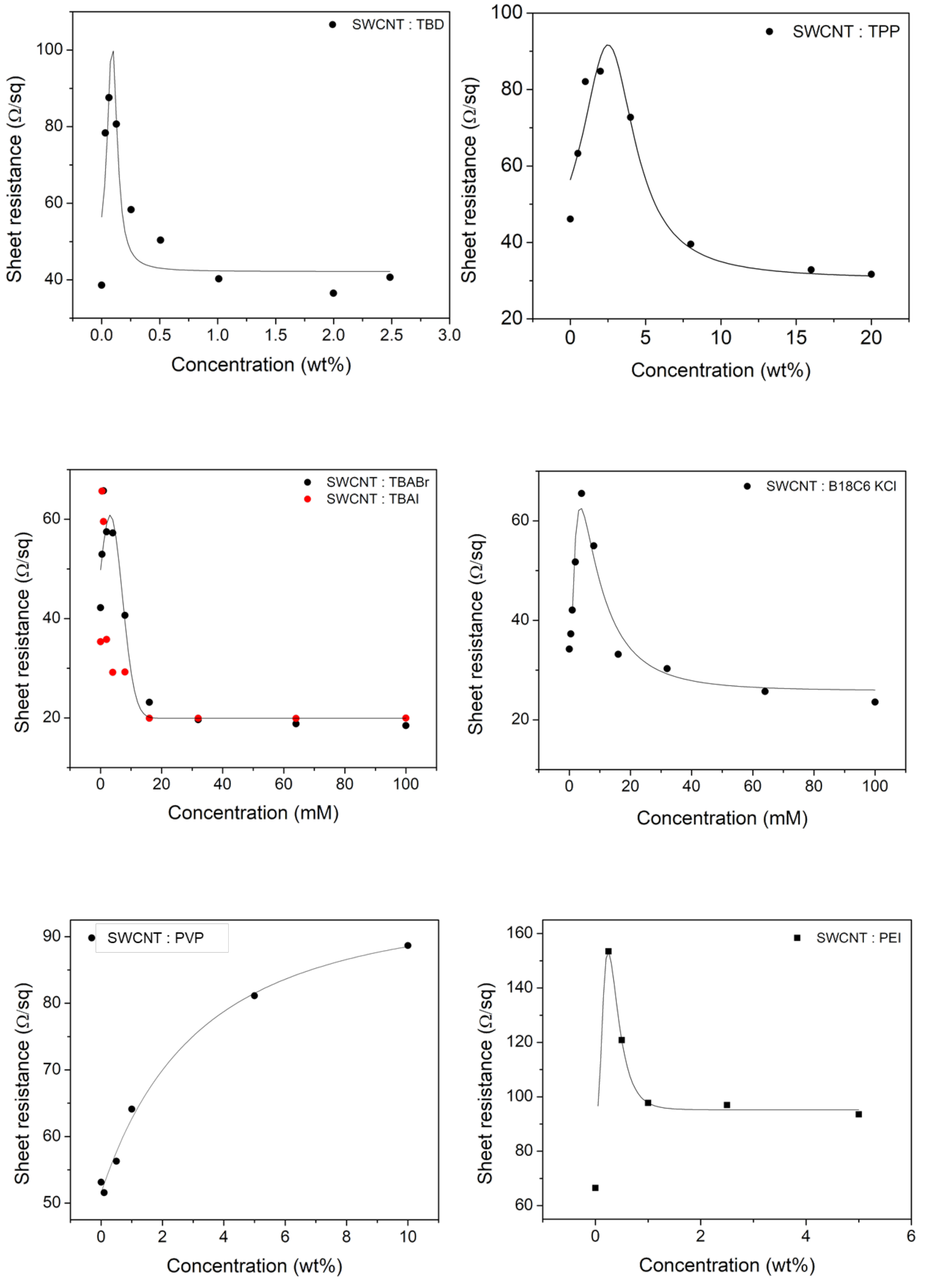

Figure S5. Sheet resistance of SWCNT films treated with various dopants at different doping concentrations.

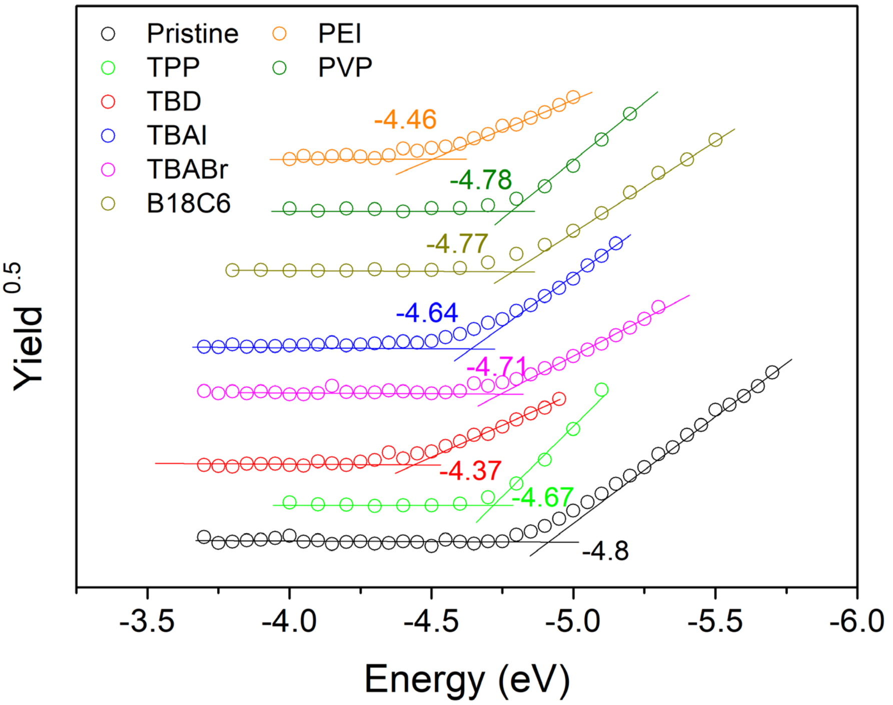

Figure S6. Photoemission yield spectra of pristine and doped SWCNT films. The work function values were determined from the threshold onset of photoemission.

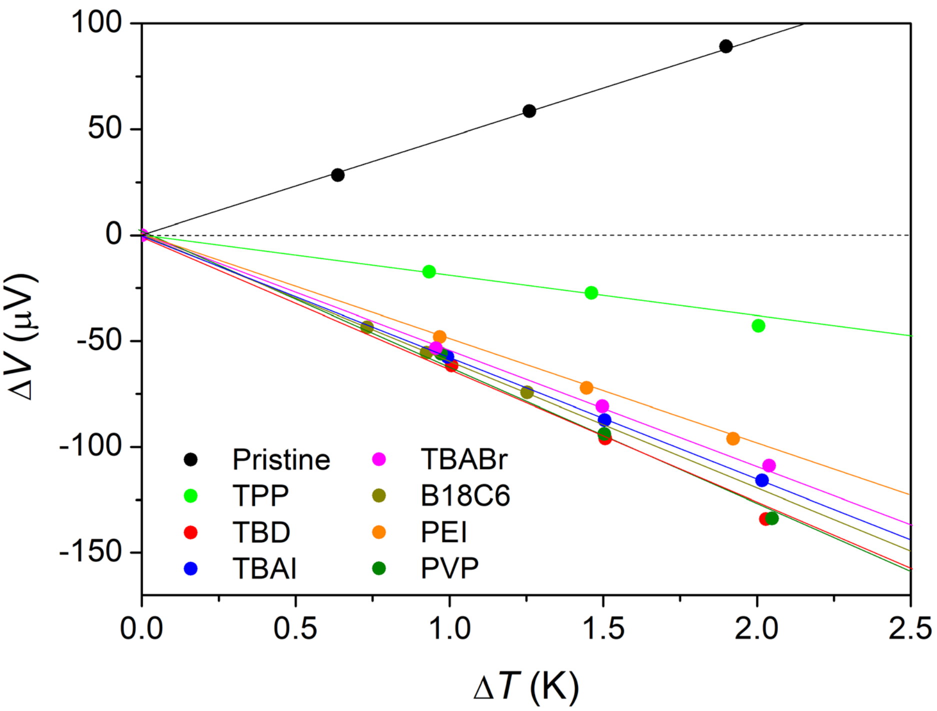

Figure S7. Thermoelectric voltage (ΔV) versus applied temperature difference (ΔT) of pristine and doped SWCNT films. Seebeck coefficients (S) were obtained from the linear slope of the $\Delta V/\Delta T$ plots and corrected for contact wire contributions from the instrument (22.7 μV K^{-1}).

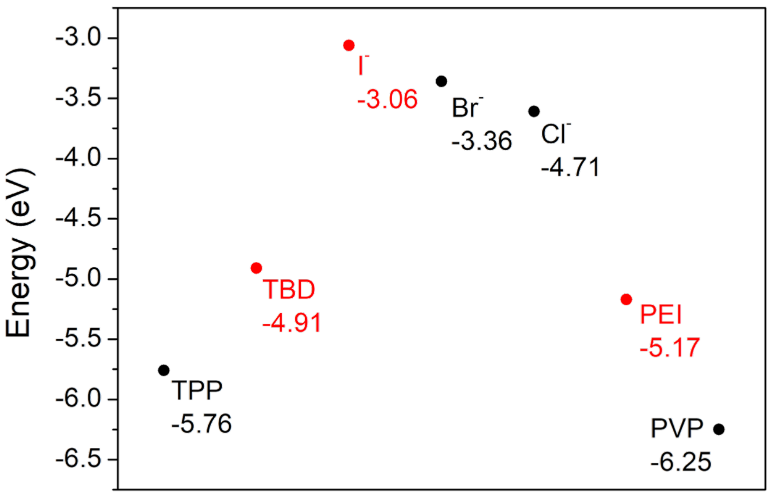

Figure S8. DFT-calculated electronic parameters of the investigated dopants, including HOMO energy levels of molecular dopants and electron affinity from ionic dopants [1]. The PEI and PVP models were represented by diethylenetriamine (DETA) and N-methyl-2-pyrrolidone (NMP) molecules, respectively, to reflect their dominant functional units and electronic characteristics.

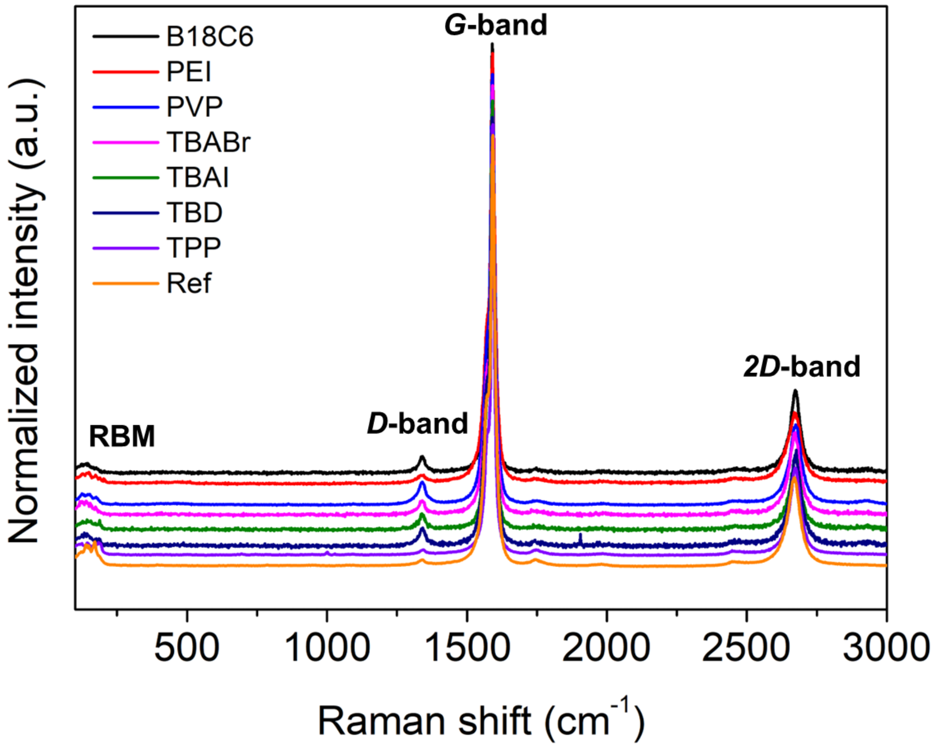

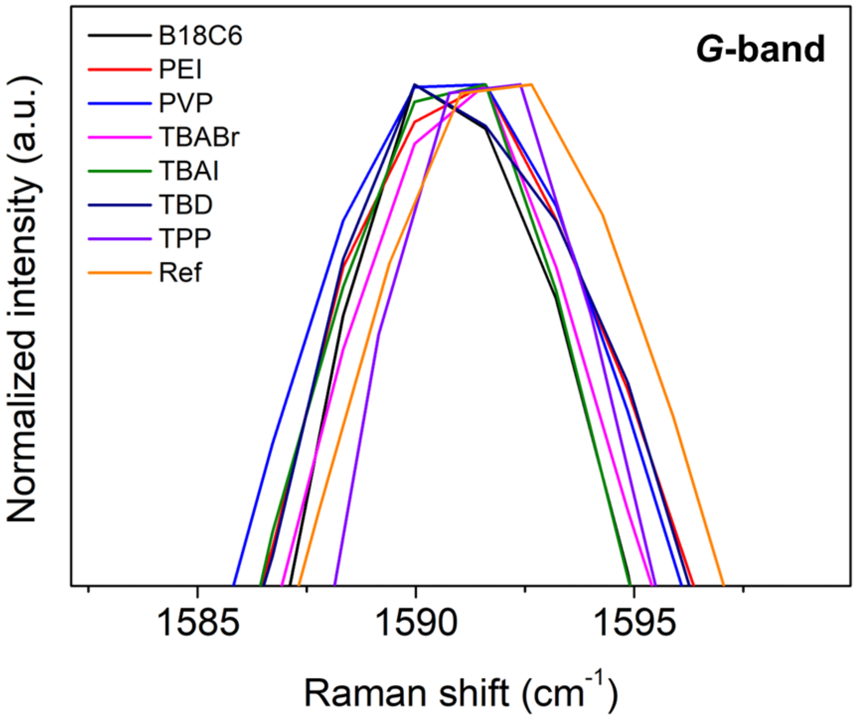

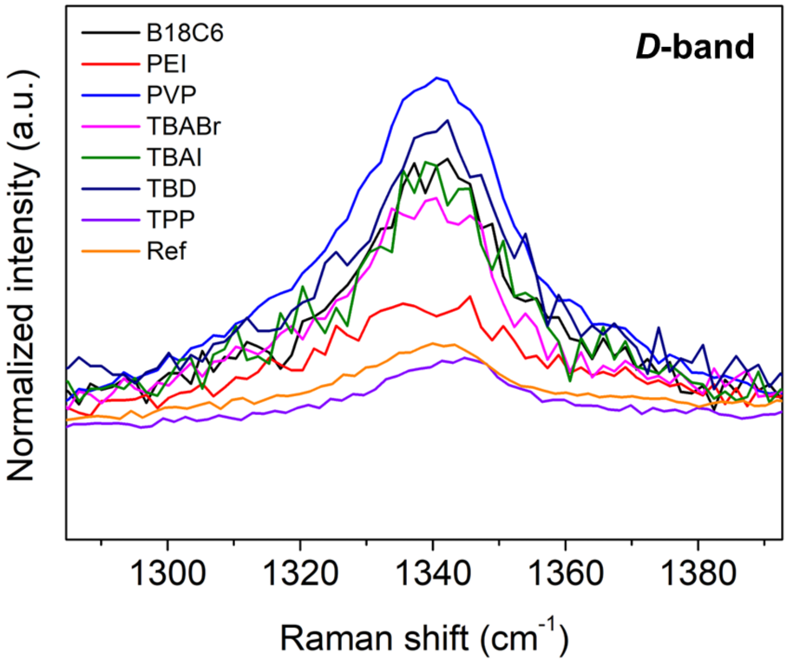

Figure S9. Raman spectra of pristine and doped SWCNT films. D- and G-band regions are shown for comparison.

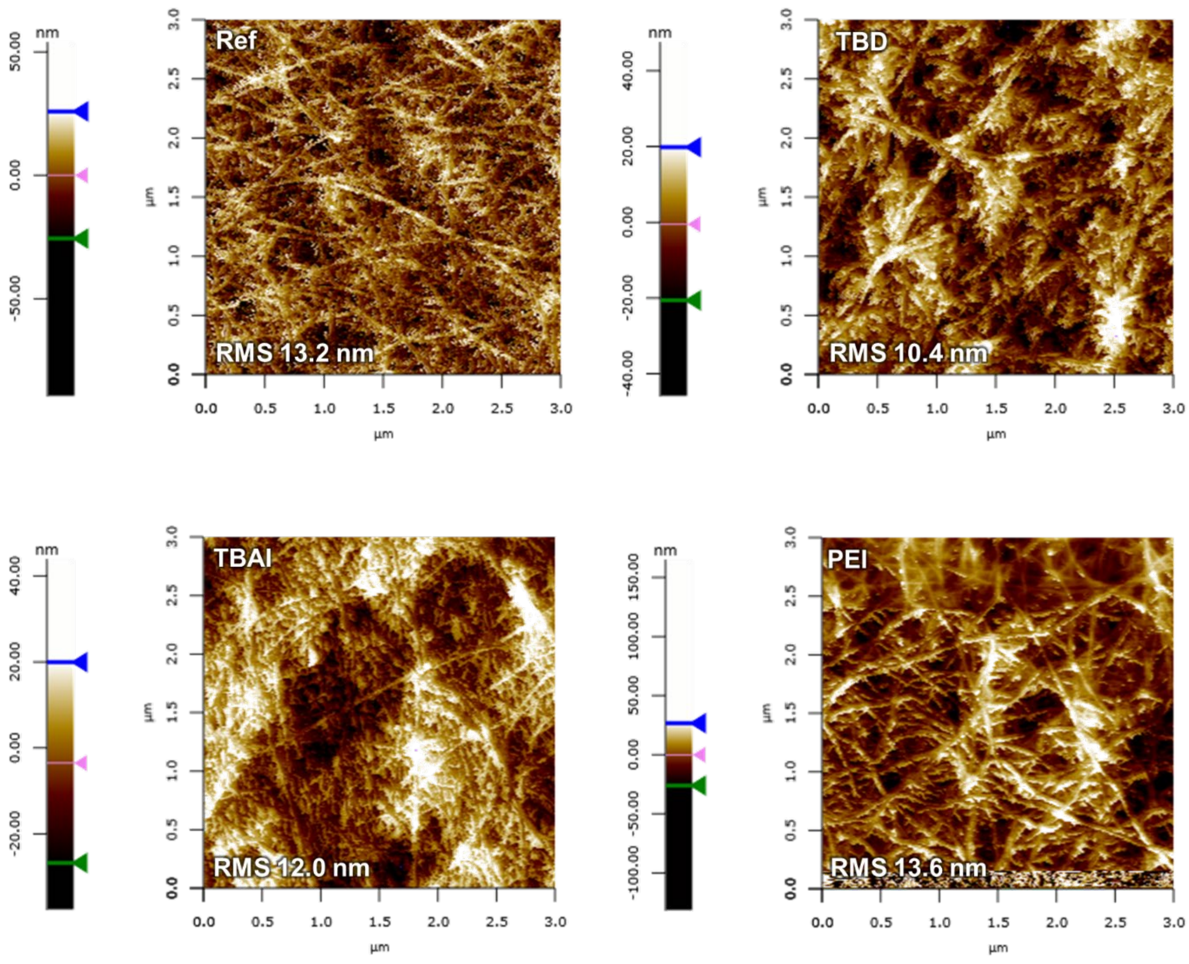

Figure S10. AFM images of pristine and doped SWCNT films (PEI, TBD, and TBAI) and their corresponding root-mean-squared roughness (RMS, R_q).

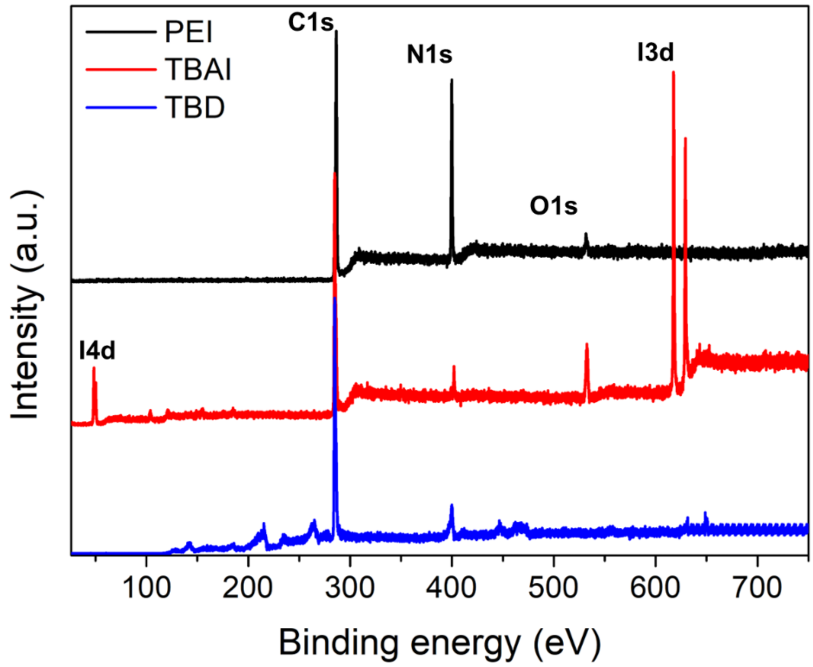

Figure S11. Survey XPS spectra of doped SWCNT films (PEI, TBD, and TBAI) labelled against their corresponding elemental compositions.

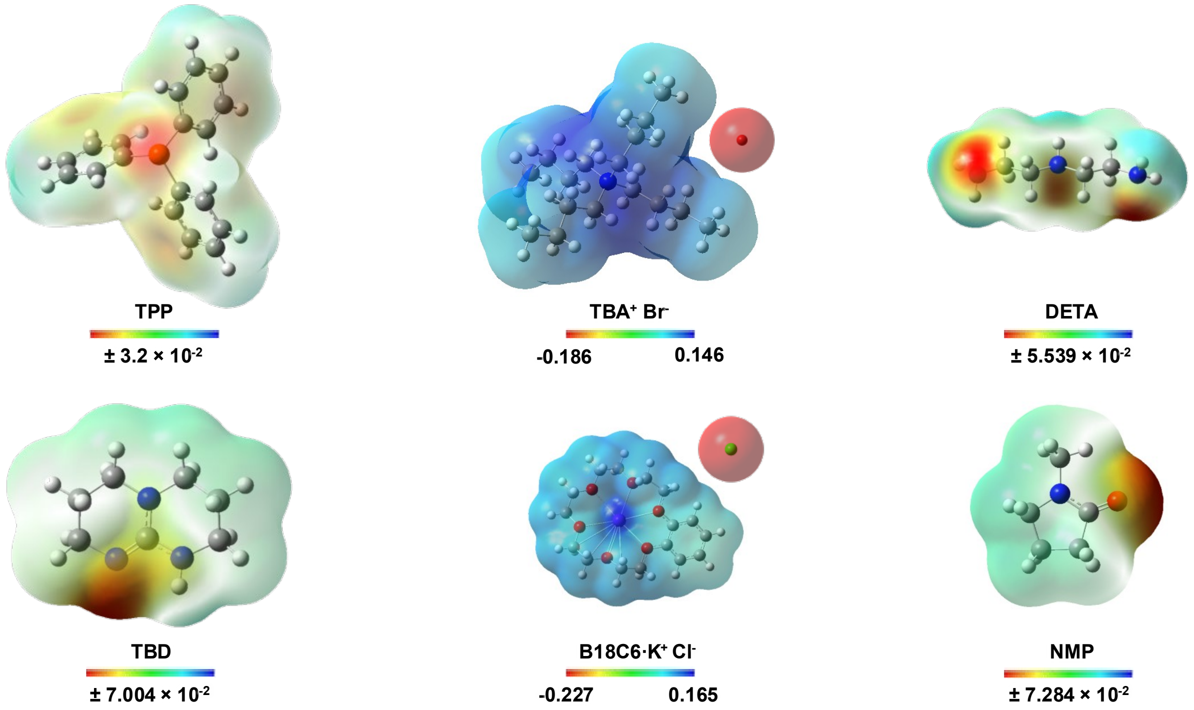

Figure S12. Molecular electrostatic potential (MESP) maps of the investigated dopants. The red and blue regions represent electron-rich and electron-deficient sites, respectively, indicating potential locations for charge transfer interactions with SWCNTs.

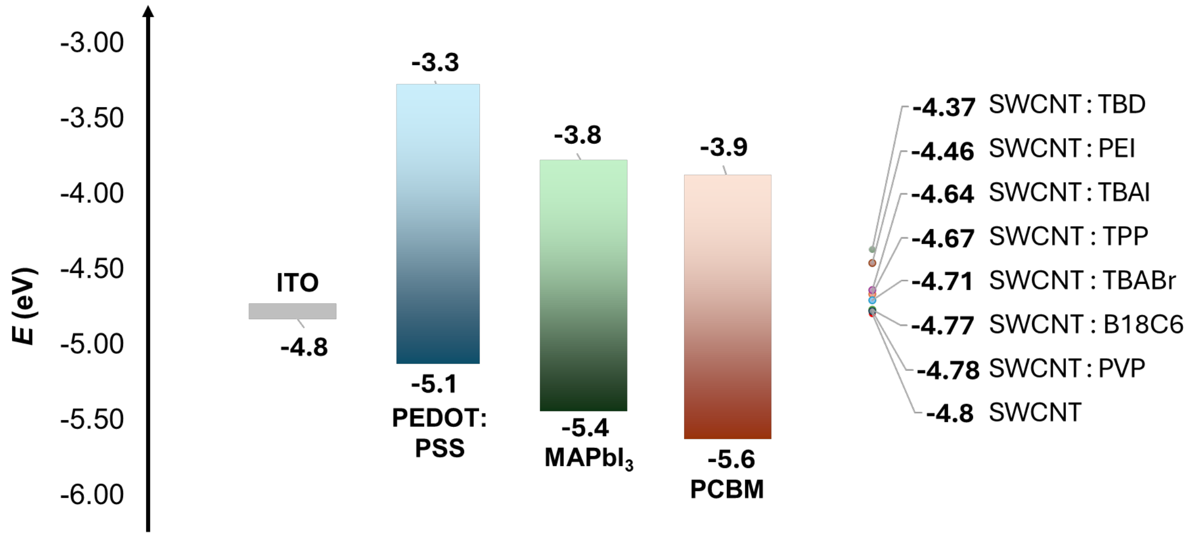

Figure S13. Energy level diagram of complete device stack incorporating pristine and doped SWCNT films.

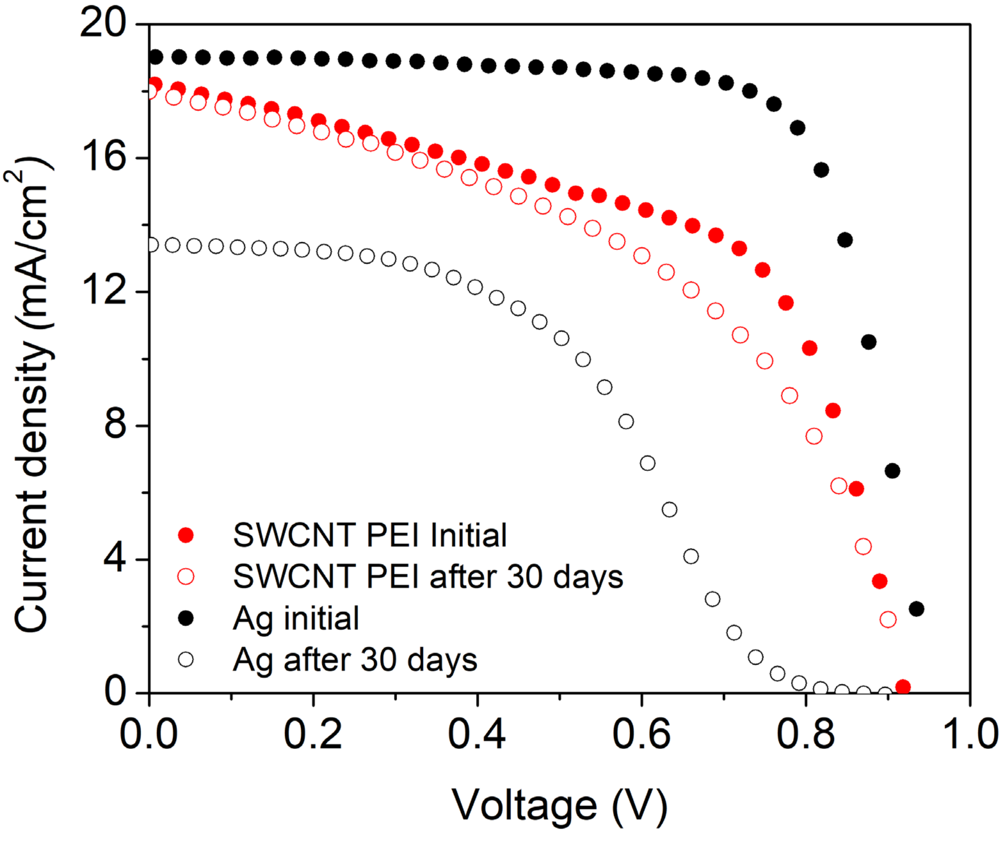

Figure S14. *J–V* characteristics comparison of inverted perovskite solar cells incorporating PEI doped SWCNT cathodes compared with silver (Ag).

Table S1. XYZ Cartesian coordinate from optimized structure of the n-type dopants.

TPP	
P -2.86520 0.28623 0.00000	C -0.10786 -1.45177 1.11190
C -2.98213 1.40057 0.00023	C -1.38210 -0.88239 1.11339
C -1.84169 -0.16968 -0.00026	H -4.41494 0.19271 1.99155
C -3.77179 -0.37221 0.00003	H -6.40126 -1.25482 1.97869
C -2.59459 2.15446 1.11417	H -6.91950 -2.65831 0.00014
C -2.73859 3.54267 1.11328	H -5.42463 -2.59973 -1.97847
C -3.27490 4.19097 0.00079	H -3.43363 -1.15864 -1.99147
C -3.66502 3.44592 -1.11197	H -1.32990 0.51723 -1.99153
C -3.51775 2.05805 -1.11342	H 0.91363 -0.48647 -1.97917
C -4.61834 -0.41401 1.11403	H 1.71200 -1.75260 -0.00116
C -5.74857 -1.23283 1.11290	H 0.23762 -2.00638 1.97742
C -6.04196 -2.02097 0.00011	H -2.00915 -1.00998 1.99092
C -5.20176 -1.98583 -1.11272	H -3.83310 1.50119 -1.99091
C -4.07347 -1.16434 -1.11393	H -4.08560 3.94599 -1.97749
C -1.00495 -0.03440 -1.11425	H -3.38808 5.26961 0.00101
C 0.27063 -0.60079 -1.11340	H -2.43093 4.11880 1.97902
C 0.72128 -1.31130 -0.00091	H -2.17050 1.67483 1.99145
TBD	
C -1.28460 1.38904 -0.24355	H -1.49269 1.53503 -1.31796
C -2.45355 -0.82911 -0.09844	H -1.17459 2.38492 0.20350
N -1.13486 -1.47602 -0.05554	H -3.14911 -1.42998 0.49991
N -0.02276 0.66380 -0.04694	H -2.83846 -0.85035 -1.13104
N 1.16058 -1.38655 0.05810	H 1.09839 2.39931 0.23047
C 1.22515 1.41221 -0.23173	H 1.43067 1.58386 -1.30426
C 2.40686 0.67394 0.40598	H 3.34039 1.20229 0.18187
C 2.46065 -0.75868 -0.13504	H 2.27940 0.64895 1.49427
H 1.07428 -2.39107 0.06100	H 2.75722 -0.73600 -1.19922
C -2.43804 0.62210 0.41585	H 3.21774 -1.34425 0.39811
H -3.39049 1.12445 0.20739	C -0.06353 -0.73634 -0.03251
H -2.29108 0.62536 1.50323	
TBABr	
N -0.00006 0.00026 0.00048	H -1.35814 -1.07781 1.22922
C 0.87541 -0.26310 -1.24748	H -1.44919 -1.76332 -1.77745
C 1.92049 0.80417 -1.58745	H -2.63587 -0.76595 -0.93081
C 2.70012 0.39882 -2.86134	H -1.99432 -3.69426 -0.26823
C 3.76454 1.43777 -3.24667	H -3.17870 -2.70164 0.57785
C -0.87572 1.24796 -0.26272	H -3.31199 -3.44791 -2.41534
C -1.92142 1.58725 0.80417	H -4.29982 -4.14963 -1.12688
C -2.70196 2.86043 0.39832	H -4.50445 -2.44811 -1.56446
C -3.76667 3.24540 1.43711	H 0.17774 0.37896 2.07986
C -0.87546 -1.24771 0.26376	H 1.35887 1.22875 1.07760
C -1.92088 -1.58749 -0.80329	H 1.44861 -1.77725 1.76593
C -2.69989 -2.86188 -0.39835	H 2.63423 -0.93331 0.76497

C -3.76451 -3.24707 -1.43716	H 1.99870 -0.26490 3.69330
C 0.87580 0.26358 1.24810	H 3.18238 0.57779 2.69697
C 1.92090 -0.80382 1.58765	H 4.30400 -1.12551 4.14616
C 2.70263 -0.39749 2.85993	H 4.50488 -1.56683 2.44515
C 3.76661 -1.43692 3.24523	H 3.31316 -2.41409 3.44890
H -4.30307 4.14708 1.12614	H 0.17701 -0.37851 -2.07896
H -4.50568 2.44575 1.56540	H 1.35850 -1.22828 -1.07719
H -3.31406 3.44767 2.41496	H 2.63516 0.93235 -0.76573
H -3.18087 2.69876 -0.57758	H 1.44852 1.77808 -1.76397
H -1.99733 3.69346 0.26724	H 1.99491 0.26802 -3.69392
H -1.44985 1.76442 1.77814	H 3.17918 -0.57712 -2.70037
H -2.63546 0.76499 0.93241	H 4.50413 1.56576 -2.44750
H -1.35826 1.07799 -1.22822	H 4.30029 1.12717 -4.14886
H -0.17753 2.07974 -0.37727	H 3.31177 2.41569 -3.44822
H -0.17692 -2.07916 0.37841	Br 1.44861 -1.77725 1.76593
B18C6·KCl	
C 3.10616 -2.66358 0.30565	H -5.90726 -1.48788 -1.03025
C 2.06286 -3.54732 -0.36255	H -6.00456 1.00043 -1.19203
C 0.01935 -3.03956 0.89201	H -4.05800 2.35636 -0.50792
C -1.43940 -2.72916 0.62578	H -2.07483 -3.13256 1.42689
C 4.37853 -0.62511 -0.04078	H -1.72869 -3.18380 -0.32854
C 4.25107 0.71728 -0.73065	H 0.10427 -4.04216 1.34445
C 3.01206 2.80580 -0.57368	H 0.42782 -2.30032 1.59146
C 1.97523 3.49355 0.28992	H 2.07090 -4.53577 0.12911
C -0.36951 3.29034 0.88145	H 2.32787 -3.67384 -1.41465
C -1.69420 2.79250 0.34801	H 4.04362 -3.24108 0.39051
C -2.84378 0.66298 0.03979	H 2.80145 -2.37503 1.32337
C -2.78157 -0.75132 0.11375	H 4.31379 -0.47252 1.04584
C -3.89096 -1.50934 -0.26602	H 5.35340 -1.08491 -0.27122
C -5.05649 -0.88219 -0.73625	H 3.90496 0.56619 -1.76243
C -5.11146 0.50582 -0.82482	H 5.23122 1.22248 -0.75978
C -4.00419 1.27729 -0.43510	H 3.92924 3.41707 -0.61904
O 0.71598 -3.01710 -0.38138	H 2.62085 2.68681 -1.59351
O 3.30902 -1.48672 -0.50820	H 2.02951 4.58648 0.15741
O 3.30620 1.51795 0.02190	H 2.18181 3.25971 1.34365
O 0.66047 3.01802 -0.09660	H -0.14066 2.79160 1.83343
O -1.71473 1.34542 0.46967	H -0.44637 4.37563 1.06550
O -1.58691 -1.28365 0.57429	H -1.80950 3.08929 -0.70158
H -3.85872 -2.58984 -0.20339	H -2.51455 3.22838 0.93623
K 1.97523 3.49355 0.28992	Cl 4.50413 1.56576 -2.44750
DETA	
N -3.70127 -0.26566 -0.06644	H -1.39150 -1.03212 1.02312
N 0.00173 0.34987 0.10723	H 1.20449 -1.10574 -0.79324
N 3.69772 -0.26493 -0.10223	H 1.39798 -1.05662 0.96233
C -2.45962 0.50827 -0.04501	H 2.41279 1.29282 0.66903
C -1.24675 -0.41781 0.11494	H 2.36490 1.01363 -1.07400

C 1.24497 -0.42283 0.06247	H 0.01846 1.05997 0.83775
C 2.45504 0.50304 -0.10978	H -4.54113 0.24522 0.17147
H -2.50675 1.18924 0.81580	H -3.82664 -0.85438 -0.88140
H -2.29420 1.13642 -0.93813	H 3.93290 -0.70898 0.77688
H -1.21378 -1.11587 -0.73358	H 4.49341 0.17748 -0.54161
NMP	
C 1.81093 -0.49942 -0.17721	H 0.47493 -2.21704 -0.60031
C 1.32836 0.93781 0.11995	H 2.67524 -0.79482 0.42293
C -0.19324 0.86813 0.00266	H 2.09102 -0.59165 -1.23201
C 0.57367 -1.39133 0.11413	H 1.72763 1.68856 -0.56618
N -0.55344 -0.46081 -0.01196	H 1.58316 1.25966 1.13802
C -1.93788 -0.90155 -0.02210	H -2.12990 -1.57252 -0.86842
O -0.98547 1.83013 -0.04398	H -2.56741 -0.01378 -0.11404
H 0.60688 -1.82644 1.12482	H -2.19481 -1.42921 0.90622

Table S2. Electrical properties of inverted PSCs utilizing PEI doped SWCNT film compared with Ag as cathode.

**Cathode**	**Measuring time**	J_{SC} **(mA/cm^2)**	V_{OC} **(V)**	**FF**	R_S **(Ω·cm^2)**	R_{Sh} **(Ω·cm^2)**	η **(%)**
PEI doped SWCNT	Initial	18.23	0.920	0.570	7.86	204.31	9.56
	After 30 days	17.99	0.926	0.477	5.04	165.59	7.95
Ag	Initial	19.03	0.954	0.739	7.24	7170.3	13.41
	After 30 days	13.41	0.864	0.459	19.61	1382.7	5.33

Table S3. D-to-G band intensity ratio (I_D/I_G) of pristine and dopant-treated SWCNT films extracted from Raman spectra.

**SWCNT dopants**	I_D/I_G
Pristine Ref	0.007
TPP	0.01134
TBD	0.04794
TBAI	0.04188
TBABr	0.03669
B18C6	0.04239
PEI	0.02239
PVP	0.05414

Reference for supporting information

1. Hotop, H.; Lineberger, W.C. Binding Energies in Atomic Negative Ions. *J. Phys. Chem. Ref. Data* **1975**, *4*, 539–576, doi:10.1063/1.555524.
